# Volumetric Parameters Derived from CXCR4-Directed PET/CT Predict Outcome in Patients with Gastrointestinal Neuroendocrine Carcinomas

**DOI:** 10.1007/s11307-024-01899-w

**Published:** 2024-02-08

**Authors:** Kerstin Michalski, Wiebke Schlötelburg, Philipp Hartrampf, Marieke Heinrich, Sebastian Serfling, Andreas K. Buck, Rudolf A. Werner, Aleksander Kosmala, Alexander Weich

**Affiliations:** 1 Department of Nuclear Medicine, University Hospital Würzburg, 97080 Würzburg, Germany; 2 European Neuroendocrine Tumor Society (ENETS) Center of Excellence, NET Zentrum, University Hospital Würzburg, 97080 Würzburg, Germany; 3 The Russell H. Morgan Department of Radiology and Radiological Science, Division of Nuclear Medicine and Molecular Imaging, Johns Hopkins University School of Medicine, Baltimore, MD 21218 USA; 4 Department of Nuclear Medicine, Clinic for Radiology and Nuclear Medicine, University Hospital, Goethe University Frankfurt, 60596 Frankfurt, Germany; 5 Department of Internal Medicine II, Gastroenterology, University Hospital Würzburg, 97080 Würzburg, Germany

**Keywords:** CXRC4-directed PET/CT, Neuroendocrine carcinoma, Volumetric parameters, Outcome prediction

## Abstract

**Background:**

Gastro-entero-pancreatic neuroendocrine carcinomas (GEP-NECs) are an aggressive subgroup of neuroendocrine neoplasms (NENs). In patients affected with NEN, there is a growing body of evidence that increased C-X-C motif chemokine receptor (CXCR4) expression is linked to decreasing overall survival (OS) in an ex-vivo setting. Thus, we aimed to determine whether the *in-vivo*-derived CXCR4-directed whole-body PET signal can also determine GEP-NEC patients with shorter OS.

**Methods:**

We retrospectively included 16 patients with histologically proven GEP-NEC, who underwent CXCR4-directed PET/CT for staging and therapy planning. We assessed maximum, peak, and mean standardized uptake values as well as whole-body tumor volume (TV) and total-lesion uptake (TLU = SUVmean × TV) using a semi-automatic segmentation tool with a 50% threshold. Association of PET-based biomarkers and OS or radiographic progression-free survival (rPFS; according to RECIST 1.1 criteria) was analyzed using univariable and multivariable cox regression.

**Results:**

Median OS and rPFS was 7.5 and 7 months, respectively. A significant correlation between TV and TLU was found for OS (TV: hazard ratio (HR) 1.007 95% confidence interval (CI) 1.000–1.014, *p* = 0.0309; TLU: HR 1.002 95% CI 1.000–1.003, *p* = 0.0350) and rPFS (TV: HR 1.010 95% CI 1.002–1.021; *p* = 0.0275; TLU: HR 1.002 95% CI 1.000–1.004, *p* = 0.0329), respectively. No significant correlation with OS or rPFS was found for non-volumetric parameters (*p* > 0.4). TV remained a significant predictive marker for OS and rPFS in multivariable analysis (OS: HR 1.012 95%, CI 1.003–1.022, *p* = 0.0084; rPFS: HR 1.009, 95% CI 0.9999–1.019, *p* = 0.0491), whereas TLU remained only prognostic for OS (HR 1.009, 95% CI 0.9999–1.019, *p* = 0.0194) but narrowly failed significance for rPFS (*p* = 0.0559).

**Conclusion:**

*In-vivo* assessment of CXCR4 PET-derived volumetric parameters is predictive for outcome of patients with GEP-NEC and could be used as a risk stratification tool, which detects patients prone to early progression.

## Introduction

Gastro-entero-pancreatic neuroendocrine carcinomas (GEP-NECs) are highly aggressive neuroendocrine neoplasms (NENs). The differentiation of NEN is defined upon histomorphological features and proliferation index (Ki-67) [1]. NECs are dedifferentiated neoplasms that constitute only 10–20% of all NENs and are associated with a poor prognosis [2]. Whereas treatment of well-differentiated NEN is based upon targeting the somatostatin receptor (SSTR) using somatostatin analogs or SSTR-directed radioligand therapy [3], treatment possibilities of NECs are largely limited to conventional chemotherapy because of a lack of SSTR expression and a highly aggressive tumor growth [4]. Both European Neuroendocrine Tumor Society (ENETS) and European Society for Medical Oncology (ESMO) guidelines recommend platinum-based chemotherapy using cisplatin/etoposide or carboplatin/etoposide as first-line therapy for advanced NEC [3, 5]. However, there is no standardized treatment regime for second-line therapy and proposed therapies include fluorouracil-based chemotherapy together with either irinotecan or oxaliplatin as well as temozolomide in monotherapy or in combination with capecitabine. Nevertheless, progression is unavoidable [4] and further therapeutic options or personalized treatments are therefore desperately warranted.

A factor that might influence outcome of patients affected with NECs is the expression of C-X-C motif chemokine receptor (CXCR4). CXCR4 is a G-protein-coupled receptor which is overexpressed in multiple hematological malignancies and solid cancers and a possible target for theranostic approaches [6]. Our study group has shown that CXCR4-targeted imaging using the radiopharmaceutical [^68^Ga]Ga-PentixaFor is an effective image tool in staging a variety of neoplasms, especially multiple myeloma, mantle cell lymphoma, small cell lung cancer, and adrenocortical neoplasms [7]. Of note, the therapeutic counterpart [^177^Lu]Lu/[^90^Y]Y-PentixaTher can be used for CXCR4-directed radioligand therapy and has proven substantial anti-lymphoma effect in selected malignancies [8–10]. In an earlier work of our study group, we assessed the diagnostic value of CXCR4-directed PET/CT compared to the performance to reference standard [^18^F]fluorodesoxyglucose (FDG) PET/CT in patients with NEC. In this analysis of 11 treatment-naive patients, the diagnostic performance of CXCR4 PET/CT was inferior to those of FDG PET/CT and *in-vivo*-assessed CXCR4 expression was not sufficient for possible CXCR4-directed radioligand therapy. However, increased CXCR4 expression in GEP-NEN in an ex-vivo setting was linked to decreasing overall survival (OS) [11]. Of note, an ex-vivo sampling only displays tumor biology and receptor expression in the respective lesion, while assessment of the *in-vivo* CXCR4 expression offers a non-invasive whole-body evaluation, which reflects also possible intraindividual tumor heterogeneity. Thus, *in-vivo*-assessed CXCR4 expression might be a prognostic biomarker for response to therapy and outcome prediction.

The aim of this study was to investigate the prognostic impact of CXCR4 PET-derived biomarker in patients with NEC and their potential impact on OS and radiographic progression-free survival (rPFS).

## Methods

### Patient Population

Searching our institutional PET/CT database we retrospectively identified 16 patients with neuroendocrine carcinomas of the gastrointestinal tract who underwent [^68^ Ga]Ga-PentixaFor PET/CT between July, 2015, and July, 2022. Patients were also included in case of unknown primary if imaging and histopathological results indicated gastrointestinal origin. Patients were excluded if neither follow-up imaging nor survival data was available. Patients were referred to our institution by the treating physicians to evaluate potential theranostic considerations after first-line therapy using the therapeutic equivalent [^177^Lu]Lu- or [^90^Y]Y-PentixaTher [12]. In 15 of 16 patients (94%) a recent FDG PET/CT was available as part of clinical routine diagnostic. Due to the retrospective character of this study, the local institutional review board waived the requirement for additional approval (No. 20230721 01). All patients provided written informed consent. Parts of this cohort have been examined in previous studies [7, 13–18]; however, without comparing PET-based parameters to patient outcome.

### Imaging Procedure

All PET/CTs were performed on Siemens Biograph mCT 64 or 128 scanners (Siemens Healthineers, Erlangen, Germany). PET/CTs were acquired approximately 60 min after the administration of 115 ± 29 MBq [^68^ Ga]Ga-PentixaFor or 292 ± 30 MBq [^18^F]FDG from vertex to mid-thigh. The acquisition parameters were 2–3 min/bed position (mCT 64)/continuous bed motion at 1.1 mm/s (mCT 128); 3 iterations, subsets 24 (mCT 64)/21 (mCT 128); matrix, 200 * 200; Gaussian filter, 2.0 mm. A contrast-enhanced diagnostic CT scan or a low dose CT scan was acquired for attenuation correction and anatomical coregistration (tube voltage, 100–120 kV; tube current modulation; reconstructed axial slice thickness 3.0–5.0 mm) [15, 18].

### Image Evaluation

Images were analyzed by one reader (KM). Fiji [19] and the Beth Israel plugin [20] were used for autosegmentation of PET-positive lesions, i.e., volumes of interest (VOIs). VOIs including tissue with physiological radioligand uptake were removed manually, while VOIs for pathological lesions not detected by autosegmentation were added manually by the reader. In analogy to whole-body tumor segmentation on prostate-specific membrane antigen-targeted PET/CT [21, 22], we used a lesion-specific threshold of 50% of the local SUVmax for CXCR4 PET as well as for FDG PET according to the guideline of the European Association of Nuclear Medicine (EANM) [23]. The summed volumes of all lesions represent the whole-body tumor volume (= TV, measured in milliliters). In addition, we assessed maximum, peak, and mean standardized uptake values as well as total-lesion uptake (= SUVmean × TV). Radiographic PFS was assessed by two board-certified radiologists in consensus (AK and WS) using RECIST 1.1 criteria on CT follow-up imaging [24].

### Statistical Analysis

Statistical analyses were performed using GraphPad Prism Version 9.3.1 (GraphPad Prism Software, La Jolla, CA, USA). Descriptive data are presented as mean ± standard deviation or median and range. A paired *t*-test was used to assess differences between the two PET tracers. Outcome data is analyzed using univariable and multivariable cox regression including age, Ki67-score, presence of liver metastases, and pretreatment (yes vs. no). OS and rPFS are calculated from the day of CXCR4 PET/CT and are presented as median with the 95% confidence interval in square brackets. A *p* value less than 0.05 was considered statistically significant.

## Results

### Patients’ Characteristics

Mean patient age was 65 ± 10 (range 44–76) years (Table 1). Most patients were male (13 of 16; 81%). Median Ki67-score was 80 (range 45–90)%. Most patients were treatment naïve (12 of 16; 75%) at the time of CXCR4 PET/CT. Mean time between initial diagnosis and CXCR4 PET was 9 ± 9 (range 2–23) months for those patients (4 of 16) who were pretreated. Median time interval between CXCR4 and FDG PET (15 of 16 patients; 94%) was 6 days (range 1–53 days). Assessment of rPFS was possible in 14 of 16 patients (88%), because follow-up imaging was not available in 2 of 16 patients (12%).

**Table 1:** Patients’ characteristics

	All patients (*n* = 16)
Age [years] mean ± SD	65 ± 10 (44–76)
Ki67 [%] median	80 (45–90)
Gender	Male: 13; female: 3
Primary tumor	
CUP	6
Stomach	4
Pancreas	3
Biliary tract	1
Esophagus	1
Rectum	1
Pretreatment	
None	12
Carboplatin/etoposide	4
FOLFIRI + bevacizumab (second line)	1
FOLFOX + bevacizumab (second line)	1
Treatment after CXCR4 PET	
Carboplatin/etoposide	14
SIRT	1
Primary surgery	3
Metastatic sites	
Lymph nodes	14
Liver	12
Bone	7
Lung	7

### CXCR4 PET-Derived Volumetric Parameters Are Prognostic for OS and rPFS

Mean parameters for TV, TLU, SUVmax, SUVpeak, and SUVmean were 83.0 ± 112.1 ml (range 0–476.6 ml), 391.3 ± 443.7 (range 0–2215.5), 9.6 ± 5.0 (range 0–18.5), 7.3 ± 4.3 (range 0–18.5), and 4.5 ± 2.3 (range 0–8.2), respectively. Of note, 2 of 16 patients (13%) did not show any pathological uptake on CXCR4 PET, despite liver and lymph node metastases seen on corresponding FDG PET/CT. Disease progression occurred in all patients. Thirteen of 16 patients (81%) died during follow-up. Median OS and rPFS of the whole patient cohort was 7.5 (*n* = 16) and 7 (*n* = 14) months, respectively. A significant correlation between TV and TLU was found for OS (TV: hazard ratio (HR) 1.007, 95% confidence interval (CI) 1.000–1.014, *p* = 0.0309; TLU: HR 1.002, 95% CI 1.000–1.003, *p* = 0.0350) and rPFS (TV: HR 1.010, 95% CI 1.002–1.021; *p* = 0.0275; TLU: HR 1.002, 95% CI 1.000–1.004, *p* = 0.0329), respectively. No significant correlation with neither OS nor rPFS was found for non-volumetric parameters (*p* > 0.4, respectively).

### FDG PET-Derived Parameters Are Not Prognostic for OS and rPFS

**Fig. 1:**
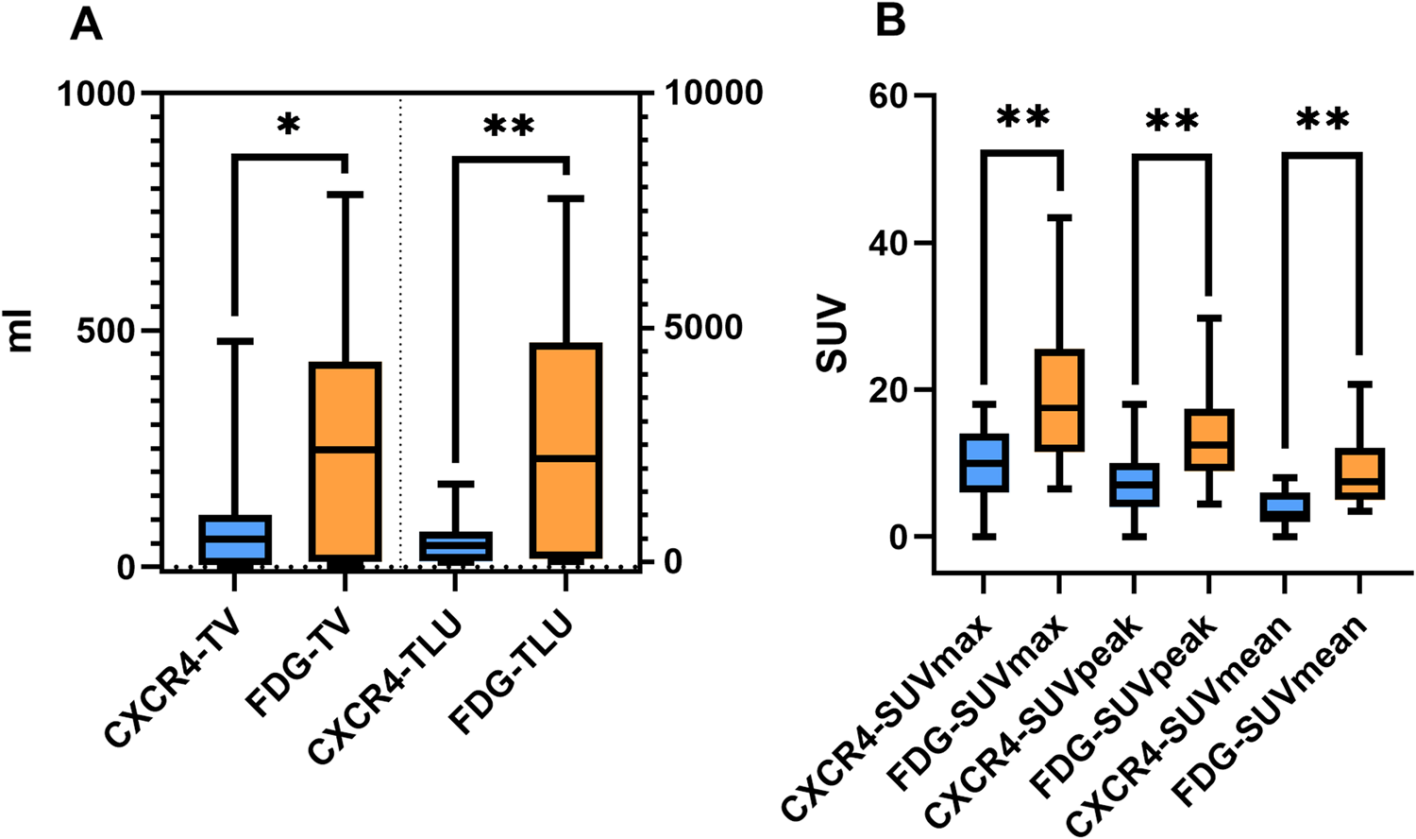
Paired *t*-test of volumetric parameters (**A**) and standardized uptake values (SUV; **B**) of CXCR4 (blue bars) and FDG (orange bars) PET derived parameters. The scale bars indicate the 5th to 95th confidence interval. Abbreviations: TV, tumor volume; TLU, total-lesion uptake (= TV * SUVmean)

**Fig. 2:**
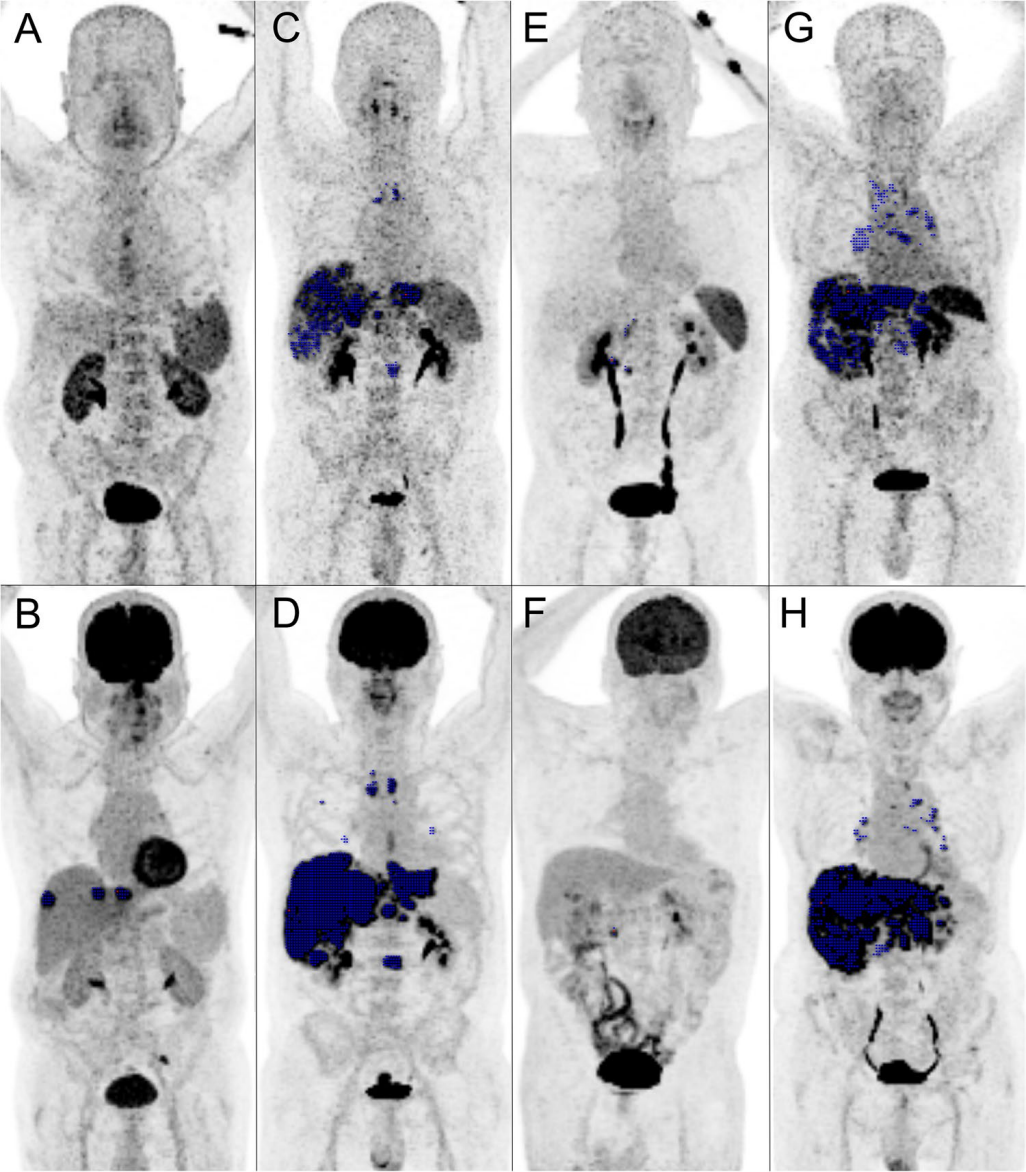
Comparison of PET-based biomarker of corresponding maximum intensity projections of CXCR4 (upper row) and FDG (lower row) PET. Tumor is delineated using Fiji [19] and the Beth Israel Plugin [20] and marked in blue. SUV window ranging from 0 to 5. **A**, **B** A 75-year-old patient with pretreated gastric neuroendocrine carcinoma (NEC) and a negative CXCR4 PET scan with missing tracer uptake of the liver metastases. **C**, **D** A 55-year old patient with initial diagnosis of an esophageal NEC and metastases in lymph nodes, liver, bones, and the lungs. **E**, **F** A 68-year-old patient with initial diagnosis of a pancreatic NEC and local lymph node metastases only seen on CXCR4 PET. **G**, **H** A 76-year-old patient with initial diagnosis of a gastric NEC and metastases in lymph nodes, liver, bones, and the lungs

Mean parameters for TV, TLU, SUVmax, SUVpeak, and SUVmean were 243.7 ± 229.0 ml (range 1.8–785.9 ml), 2422.4 ± 2510.7 (range 8.7–7758.4), 18.6 ± 10.7 (range 6.5–43.3), 14.0 ± 7.9 (range 4.5–29.7), and 9.0 ± 4.8 (range 3.5–20.8), respectively. The two patients with negative CXCR4 PET showed intense uptake on FDG PET (TV: 18.4 ml, TLU: 164.1, SUVmax: 14.7, SUVpeak: 10.3, SUVmean: 8.9 and TV: 433.9 ml, TLU: 7341.6, SUVmax: 37.1, SUVpeak: 29.7, SUVmean: 16.9). All PET-based parameter were significantly higher FDG PET compared to CXCR4 PET (*p*-values: TV: 0.0467, TLU: 0.0097, SUVmax: 0.0075, SUVpeak: 0.0082, SUVmean: 0.0046; Fig. 1). Median OS and rPFS of those patients who underwent an additional FDG PET/CT was 6 (*n* = 15) and 5.5 (*n* = 13) months, respectively. None of the volumetric or non-volumetric parameters were prognostic for OS or rPFS (*p* > 0.06). Table 2 summarizes the outcome data of CXCR4 and FDG PET-derived parameters. Figure 2 depicts different patterns of tracer uptake on CXCR4 and FDG PET.

**Table 2:** Univariable cox regression of OS and rPFS of CXCR4 and FDG PET derived parameters

	CXCR4 PET				FDG PET			
	OS		rPFS		OS		rPFS	
	HR	*p*	HR	*p*	HR	*p*	HR	*p*
TV	1.007	0.0309*	1.010	0.0275*	1.002	0.1370	1.001	0.4431
TLU	1.002	0.0350*	1.002	0.0329*	1.000	0.0855	1.000	0.6134
SUVmax	1.011	0.8600	1.017	0.8086	1.055	0.0632	1.044	0.1872
SUVpeak	1.029	0.7325	1.065	0.5266	1.070	0.0674	1.048	0.2287
SUVmean	0.9032	0.3835	0.9118	0.4459	1.126	0.0634	1.084	0.3186

Abbreviations: OS, overall survival, rPFS, radiographic progression-free survival, TV, tumor volume, TLU, total-lesion uptake, SUV, standardized uptake value

### CXCR4 PET-Derived Tumor Volume Remains a Prognostic Marker in Multivariable Cox Regression

Multivariable cox regression including age, Ki67-score, presence of liver metastases, and pretreatment (yes vs. no) for CXCR-derived TV and TLU confirmed TV as a significant predictive marker as well as for OS and for rPFS (OS: HR 1.012 95% CI 1.003–1.022, *p* = 0.0084; rPFS: HR 1.009 95% CI 0.9999–1.019, *p* = 0.0491), whereas TLU remained only predictive for OS (HR 1.009 95% CI 0.9999–1.019, *p* = 0.0194) but narrowly failed significance for rPFS (*p* = 0.0559). The other parameters were not significantly associated with OS or rPFS (*p* > 0.07, respectively).

## Discussion

This is the first study to evaluate the prognostic impact of whole-body *in-vivo* assessment of CXCR4 expression using CXCR4 PET/CT in patients with GEP-NEC. CXCR4-based TV and TLU were significantly correlated to OS and rPFS as well as in univariable and for TV also in multivariable analysis. Interestingly, corresponding FDG PET-derived biomarkers were not associated with outcome. In contrast, Langen Stokmo et al. found TV, TLU, and SUVmax on FDG PET to be significant predictors for OS and rPFS in 14 patients with gastroenteropancreatic neuroendocrine tumors G3 and 52 patients with gastroenteropancreatic NEC [25]. Jiang et al. showed a significant association between NECs of the uterine cervix in 22 patients and the volume as well as the lesion uptake solely of the primary tumor on FDG PET and PFS, whereas SUVmax of the primary tumor was not a prognostic marker [26]. Hou et al. also described a significant prognostic value of TV and TLU on FDG PET in 28 patients with esophageal NECs [27]. The fact that FDG PET was not a prognostic tool in our cohort could be due to the small sample size in which a statistical significant effect does not break through. CXCR4 PET-derived parameters appear to be more robust predictors for rPFS and OS with significant results even in a small study group.

Volumetric parameters (TV and TLU) on CXCR4 PET were the only predictive biomarker. This was not due to mere tumor extent since the “real” tumor volume on FDG PET significantly diverged. A possible reason might be tumor heterogeneity, which cannot be depicted by SUVmax/peak or SUVmean. Hence, CXCR4 PET might serve as a risk stratification tool which is able to detect patients prone to early progression. Consequently, CXCR4 PET/CT might help the treating physician to identify candidates in risk of early progression under first-line therapy or even single out high-risk lesions that can be targeted by a local procedure such as surgery, radiofrequency ablation, or chemoembolisation.

In the present study, we did not analyze the diagnostic performance or the theranostic potential of CXCR4 PET/CT compared to the reference standard. However, two patients showed false-negative CXCR4 PET scans in our patient cohort and the diagnostic inferiority of CXCR4 PET in patients with (neuroendocrine) gastrointestinal neoplasms has been shown before by our study group [14, 28]. Hence, other theranostic radiopharmaceuticals in this tumor entity would be desirable. One potential option is fibroblast activation protein (FAP)-targeting radioligands. FAP-directed PET/CT showed promising diagnostic performance compared to FDG PET/CT in a subcohort of seven patients with GEP-NEC in an analysis of our own study group [18]. These results encourage the development of a theranostic FAP-targeted twin [29, 30] in order to provide additional therapeutic strategies.

This study suffers from several limitations. Apart from the retrospective study design and the single-center approach, we only included a small sample size due to the rare incidence of NECs. We assessed rPFS based upon the routine follow-up imaging including conventional CT scan and/or FDG PET/CT. Imaging follow-up was thus not standardized performed but routinely undertaken after 3 months. We also assessed PET-based parameters of CXCR4 PET compared to the reference standard FDG, but corresponding FDG PET was not available in one patient. However, this is to our knowledge the only analysis, which includes a substantial sample size of GEP-NEC with dual tracer imaging.

## Conclusion

*In-vivo* assessment of CXCR4 PET-derived volumetric parameters is prognostic for patient outcome with GEP-NECs and might reflect tumor heterogeneity and aggressiveness. These parameters could be used for risk stratification in order to detect patients prone to early progression and support treatment decisions.
